# Mandibular Distraction Osteogenesis Guided by 3D Model and Monitored with Ultrasonography: A Case Report

**DOI:** 10.3390/pediatric18010006

**Published:** 2026-01-03

**Authors:** Barbora Hocková, Julien Issa, Miroslav Malček, Krzysztof Dowgierd, Rastislav Slávik, Yu-Chi Cheng, Karol Králinský, Adam Stebel

**Affiliations:** 1 Department of Maxillofacial Surgery, F. D. Roosevelt University Hospital, 974 01 Banská Bystrica, Slovakia; bhockova@nspbb.sk (B.H.); rslavik@nspbb.sk (R.S.); astebel@nspbb.sk (A.S.); 2 Department of Pediatric Surgery, Children Hospital, 974 01 Banská Bystrica, Slovakia; 3 Department of Oral Radiology & Digital Dentistry, Academic Center for Dentistry Amsterdam (ACTA), University of Amsterdam & Vrije Universiteit Amsterdam, 1081 LA Amsterdam, The Netherlands; j.i.issa@acta.nl; 4 Pediatric Department, Children Hospital, 974 01 Banská Bystrica, Slovakia; miroslav.malcek@gmail.com (M.M.); karol.kralinsky@dfnbb.sk (K.K.); 5 Faculty of Medicine, Clinic of Head and Neck Surgery for Children and Adolescents, Department of Clinical Pediatrics, University of Warmia and Mazury in Olsztyn, 10-561 Olsztyn, Poland; krzysztofdowgierd@gmail.com; 6 Department of Maxillofacial and Reconstructive Surgery for Children and Adolescents, Regional Specialist Children’s Hospital in Olsztyn, 10-561 Olsztyn, Poland; 7 Harvard School of Dental Medicine, Boston, MA 02115, USA

**Keywords:** mandibular distraction osteogenesis, pediatric maxillofacial surgery, ultrasonography, 3D printing, surgical planning, airway obstruction

## Abstract

This case report describes mandibular distraction osteogenesis (DO) in a six-year-old patient with first and second branchial arch syndrome and obstructive sleep apnea, in whom 3D surgical planning was combined with ultrasonography (US) for postoperative monitoring. The aim was to illustrate how patient-specific 3D modeling and a structured ultrasonography protocol can support safe mandibular advancement while limiting radiation exposure in a pediatric patient with complex craniofacial deformity. Preoperatively, a 3D-printed model of the mandible, generated from a cone beam computed tomography (CBCT) scan, was used to guide precise osteotomy planning and vector orientation. The surgical procedure was conducted using a Risdon approach and piezoelectric tools to ensure minimal trauma. Postoperative monitoring incorporated serial panoramic radiography and US at predefined time points to assess gap size, callus formation, and vascularity during distraction and consolidation. US identified early callus formation, progressive cortical bridging, and preserved callus vascularity, and, together with radiographic findings, guided the timing of distraction termination and distractor removal at 16 weeks. This case adds to the limited literature on pediatric mandibular DO by demonstrating the feasibility of integrating patient-specific 3D virtual planning with US-based follow-up to improve the safety, precision, and radiation-conscious management of DO in pediatric patients with complex craniofacial deformities.

## 1. Introduction

Distraction osteogenesis (DO) of the mandible is a fundamental and highly effective procedure in the reconstruction and management of mandibular deformities and deficiencies [1]. DO involves the application of controlled mechanical traction across a surgically created osteotomy site, thereby stimulating endogenous bone regeneration within the distraction gap. This gradual traction not only promotes bone elongation but also induces concurrent adaptive changes in the surrounding soft tissues. The procedure follows a well-established sequence comprising osteotomy, distractor placement, latency period, activation (distraction) phase, consolidation phase, and finally, distractor removal [2]. Among these, the consolidation phase remains a subject of clinical debate, particularly concerning its optimal duration. Accurate assessment of bone maturation is essential for determining the appropriate time for safe device removal and successful treatment outcomes [3].

Ultrasonography (USG) has gained increasing recognition in maxillofacial surgery as a non-invasive, radiation-free diagnostic modality, particularly for evaluating soft tissue lesions in the head and neck region [4]. While A-mode ultrasonography is primarily limited to pediatric and pregnant populations for sinus imaging, B-mode ultrasonography is widely adopted for assessing cervical lymph nodes due to its superior sensitivity compared to computed tomography (CT) and magnetic resonance imaging (MRI) [5]. Beyond its soft tissue applications, USG has demonstrated utility in the diagnosis of salivary gland pathology, detection of solid tumors, and real-time monitoring of mandibular DO progression [4,5].

The postoperative evaluation and monitoring of DO, especially in pediatric patients, continue to prompt discussion within the surgical community, largely driven by the need to minimize radiation exposure. In this context, Selim et al. [3] evaluated bone regeneration in adult patients undergoing mandibular DO using both CT and USG. Their study identified four distinct phases of callus maturation via USG that correlated well with CT-based assessments of tissue calcification, suggesting the potential of USG as a reliable surrogate for evaluating bone healing [3]. Bruno et al. [6] further examined the diagnostic performance of panoramic radiography versus USG in assessing the osteotomy gap and quality of new bone formation during mandibular DO. While both modalities yielded comparable measurements of the osteotomy gap, USG significantly outperformed panoramic radiography in evaluating callus maturity [6]. Despite its advantages, a key limitation in the clinical integration of USG lies in the dependency on operator expertise; many maxillofacial surgeons remain less experienced in the interpretation of sonographic images. Although CT remains a standard imaging tool, concerns regarding ionizing radiation have led to a growing preference for cone beam computed tomography (CBCT), a three-dimensional (3D) imaging modality that offers detailed anatomic visualization with substantially reduced radiation exposure [7,8].

Recent innovations in 3D imaging and additive manufacturing have further advanced the field of DO [9]. CBCT facilitates the generation of accurate 3D models of the scanned anatomical area, enabling precise preoperative planning of osteotomies and simulation of the distraction trajectory. These technologies enhance surgical accuracy, reduce intraoperative risks, and allow for the fabrication of patient-specific distractors customized to individual anatomical morphology [9]. In parallel, 3D-printed models provide valuable tactile references for intraoperative guidance and pre-surgical rehearsal.

Despite the expanding role of ultrasonography and 3D technologies in mandibular DO, clinical reports demonstrating their integrated application in patient-specific planning and longitudinal follow-up remain limited. This case report aims to present a radiation-conscious workflow that combines patient-specific 3D-printed mandibular and tooth-germ models with a structured ultrasonography protocol for perioperative management of pediatric syndromic mandibular DO. Specifically, we describe bilateral mandibular distraction in a six-year-old girl with first and second branchial arch syndrome, highlighting how 3D planning supported safe distraction vector design and how serial US examinations informed the timing of distraction and distractor removal.

## 2. Case Description

**Figure 1:**
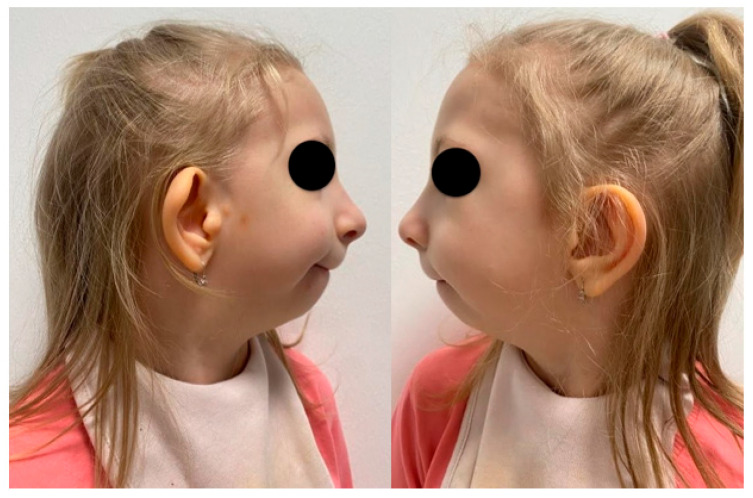
Extraoral photograph of the patient’s face from the lateral view, with significant hypoplasia of the mandible.

This case report describes mandibular DO in a six-year-old girl. The patient was born prematurely at 35 weeks of gestation, with a congenital developmental defect affecting the head and neck region (Figure 1).

Genetic examination identified the condition as first and second branchial arch syndrome. Clinically, the patient exhibited moderate obstructive sleep apnea, micrognathia, food regurgitation, and delayed eruption of permanent teeth. Surgery for a tracheoesophageal fistula was performed shortly after birth, followed by repeated esophageal dilatations to address the regurgitation. Additionally, conductive hearing loss was suspected, and at the age of five, the patient underwent surgery for excision of a branchial anomaly in the neck and extraction of carious teeth. Histological examination confirmed pilomatrixoma. At the age of six, tonsillectomy was indicated to address obstructive symptoms during sleep.

Overall health improvements were slower than anticipated, with the patient experiencing short stature for her age. Polysomnography confirmed moderate airway obstruction, and mandibular distraction was selected as a treatment to improve breathing. From the parents’ perspective, the patient’s sleep was disturbed, with interrupted sleep occurring two to three times weekly. These episodes involved waking up in the middle of the night due to shortness of breath or sensations of airway obstruction.

**Figure 2:**
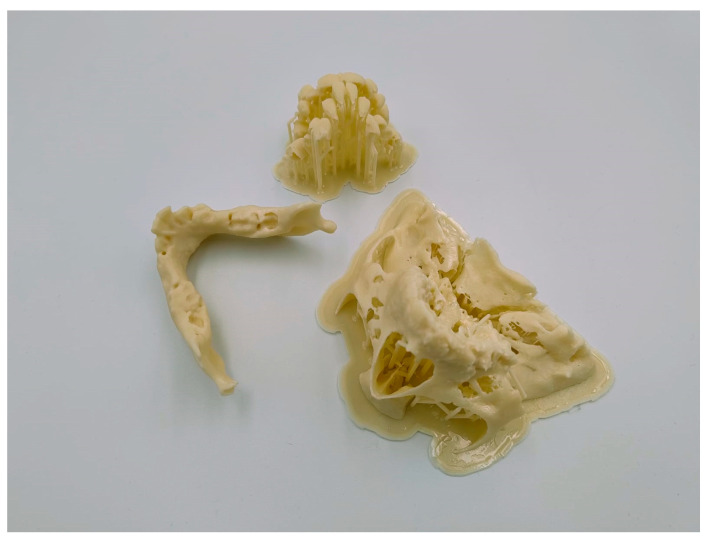
3D-printed models of the mandible, midface, and dental germs.

**Figure 3:**
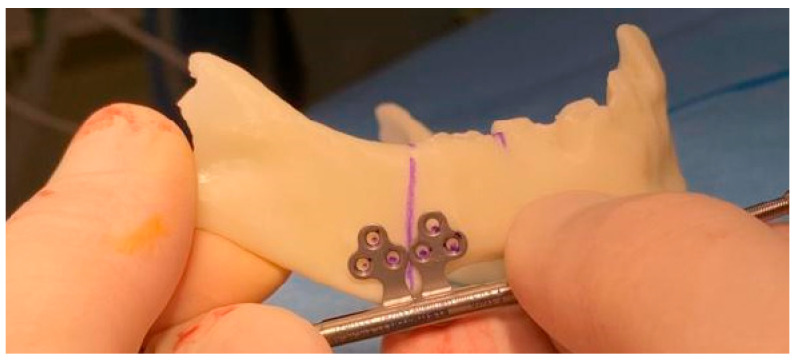
3D-printed model of the mandible with the preoperatively planned osteotomy line (purple) and distractor.

Preoperatively, a 3D surgical simulation was carried out using a patient-specific model of the mandible. CBCT of the mandible was obtained using the i-CAT with the following acquisition parameters: X-ray tube voltage of 120 kV, tube current of 5 mA, isotropic voxel resolution of 0.125 mm, and a field of view (FOV) of 4 × 4 cm. The scanned mandible was segmented using RealGUIDE software and exported in Standard Tessellation Language (STL) format. These STL files were then sent to a specialized laboratory, where a life-sized 3D model of the mandible was fabricated using an SLA 3D printer (Figure 2 and Figure 3). The CBCT-derived 3D reconstruction guided precise osteotomy planning at the mandibular angle region, with the primary goal of achieving bilateral airway expansion. To optimize osteotomy placement and avoid damage to developing dentition, an additional model illustrating the location of tooth germs was generated.

**Figure 4:**
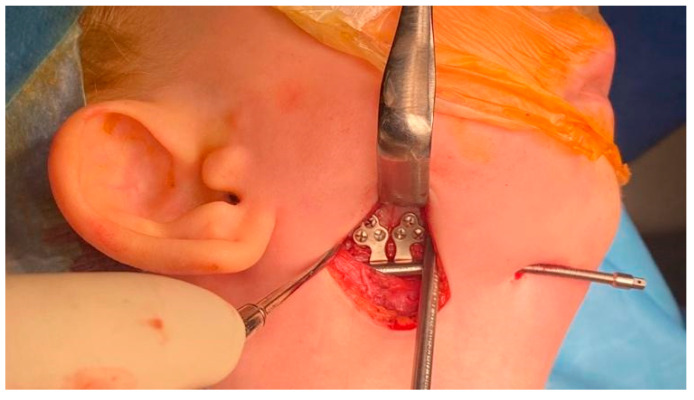
Risdon’s approach to the mandible. After osteotomy, the distractor was adapted and secured in situ.

Intubation of the patient was challenging and was performed using a video laryngoscope. After the surgical field was disinfected and draped, Risdon’s approach was used to access the corpus and angle of the mandible via the skin, subcutaneous tissue, and the platysma muscle, with a neurostimulator ensuring the protection of the marginal branch of the facial nerve. Following the incision of the periosteum, the osteotomy was carried out in alignment with the preoperative 3D model using a piezoelectric surgical tool (Figure 4).

The osteotomy was carefully performed on both the buccal and lingual surfaces of the mandible. Submental tunnels were prepared for the distractor activation arms on both sides, and KLS Martin Art. No. 51-515-15-09 distractors (KLS Martin, Jacksonville, FL, USA) were adapted and secured. The surgical wound was closed in layers with intradermal sutures.

**Figure 5:**
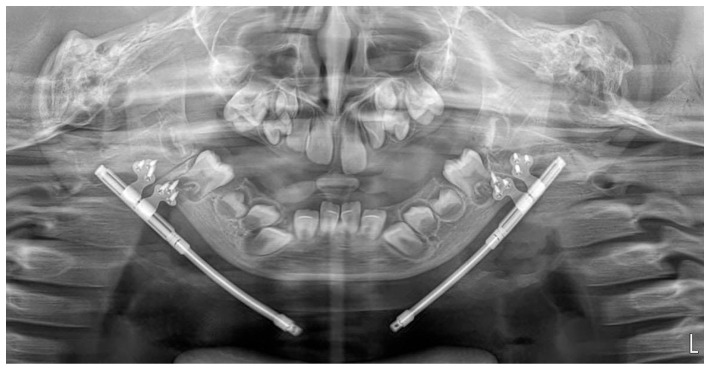
Panoramic radiograph performed on the first postoperative day showing appropriate positioning of the osteotomy line and an adapted distractor.

Postoperatively, the patient was monitored in the intensive care unit during the immediate postoperative period. She was transferred to the pediatric surgery postoperative care ward on the first postoperative day. She received amoxicillin/clavulanic acid 0.6 g intravenously every 8 h, administered according to the standard perioperative prophylactic protocol. The radiographs confirmed accurate positioning of the distractors according to the preoperative plan (Figure 5).

**Figure 6:**
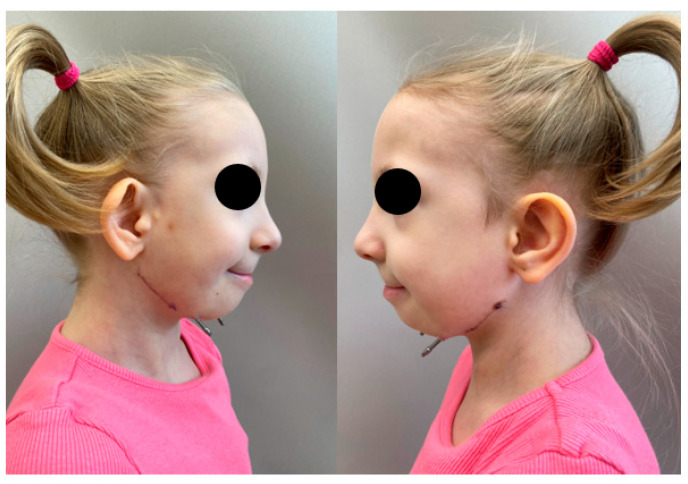
Extraoral photography of the lateral view of the patient’s face after completion of the distraction phase.

Distraction commenced on the fourth postoperative day, with two activations per day at a rate of 0.5 mm every 12 h. The goal of the distraction phase was to advance the mandibular segments to achieve an edge-to-edge occlusion, with the anticipation of minor relapse in mandibular projection during the consolidation phase due to callus remodeling and ossification. The patient was discharged on the sixth postoperative day with a prescription for penicillin administered every 8 h and was advised to adhere to a soft diet. Detailed instructions were provided to the parents regarding the distraction protocol and wound management. Specifically, they were advised to apply a broad-spectrum, non-irritating antiseptic spray to disinfect the distractor exit sites. Distraction was completed after 15 days, resulting in a total anteroposterior mandibular advancement of 15 mm (Figure 6).

Ultrasound-based monitoring was initiated on the third day of the consolidation phase to evaluate the position and symmetry of the distractors and to assess early callus formation. Examinations were performed using the ARIETTA 750 ultrasound system (Fujifilm, Lexington, USA) equipped with a 13 MHz linear transducer, without sedation. The probe was placed on the lateral aspect of the mandibular body and angle and oriented perpendicular to the cortical surface. At each examination, both the cranial and caudal cortices adjacent to the osteotomy were scanned in longitudinal and transverse planes, with particular attention to the metal distractor body, the osteotomy gap, and surrounding soft tissues. The distraction gap was measured between the echogenic cortices at the osteotomy site, and color Doppler imaging was used to assess vascularity within the developing callus. Ultrasound was performed at days 6, 21, and 42 (week 6) after surgery and again at weeks 6 and 16 after completion of distraction, in parallel with clinical and radiographic follow-up.

## 3. Results

Table 1 summarizes the key clinical and imaging findings during the mandibular distraction osteogenesis process. The data include USG measurements of the osteotomy gap, qualitative USG observations, radiographic findings, and relevant clinical findings at each stage of the distraction process.

**Figure 7:**
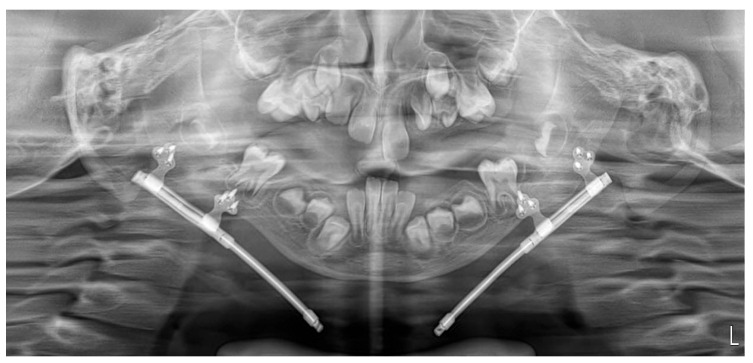
Panoramic radiograph obtained at 16 weeks after completion of distraction, immediately before removal of the distractors.

**Figure 8:**
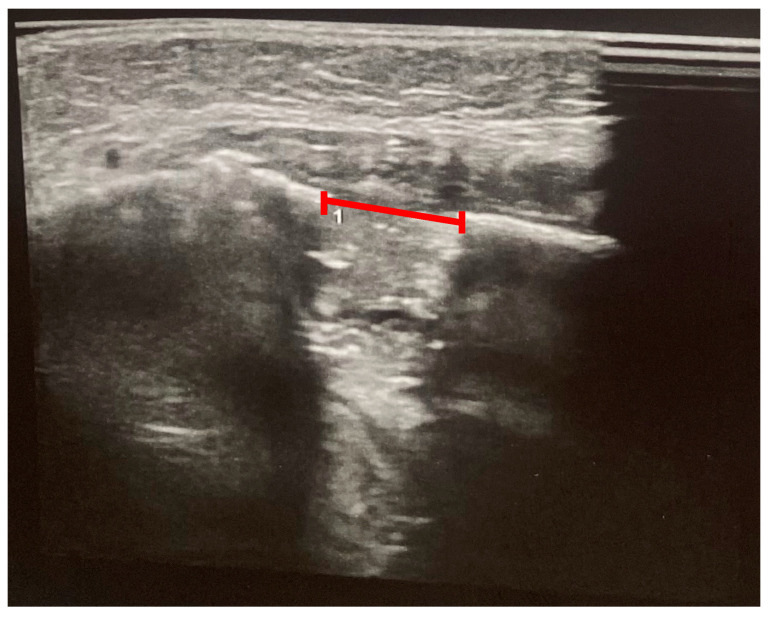
Ultrasonography at 16 weeks after completion of distraction (consolidation phase); the red line marks the residual gap between the distractors.

During the distraction and consolidation phases, we recorded the condition of the callus and its subsequent ossification by combining panoramic images and ultrasound examinations. Ultrasound examinations were performed with a 13 MHz linear transducer, without sedation. The ultrasound beam was oriented perpendicularly to the bone surface. The distractors were identified by characteristic metal echoes, and the osteotomy gap was visualized clearly, given the contrast between the gap and normal bone. Initial ultrasound findings indicated early callus formation with visible ossification. The ossification gap was measured at both cranial and caudal margins, and Doppler imaging confirmed vascularity within the callus, with no signs of infection. Further ultrasound evaluations were conducted at 6 and 16 weeks post-distraction, assessing the same parameters. Callus ossification progressed without signs of infection. At week 16, ultrasonography demonstrated a continuous, echogenic cortical line at both the cranial and caudal margins of the distraction zone, absence of a residual hypoechoic gap, and preserved intraregional Doppler signal, indicating a well-vascularized, mature callus. Panoramic radiography at the same time point confirmed cortical bridging across the distraction gap (Figure 7 and Figure 8). On the basis of these combined findings, the distraction was considered stable, and the distractors were scheduled for removal.

The second operation was performed under general anesthesia, again with the participation of an experienced anesthesia team who were prepared for difficult intubation. The aim of our procedure was to remove the distractors, which we accessed through the original scar from the Risdon approach, with excision of the scar tissue. Upon removal of the distractors, the newly formed bone displayed a distinct porous structure but had strength consistent with the surrounding bone of the original mandible. After removing the distractors, we rinsed the wounds with a disinfectant solution and closed them in anatomical layers and with intradermal sutures. During the same general anesthesia, we also performed esophagoscopy and esophageal dilation in cooperation with pediatric surgeons, indicated by the patient’s food regurgitation. The procedure was completed without complications, and the patient was discharged home on the first postoperative day.

## 4. Discussion

**Figure 9:**
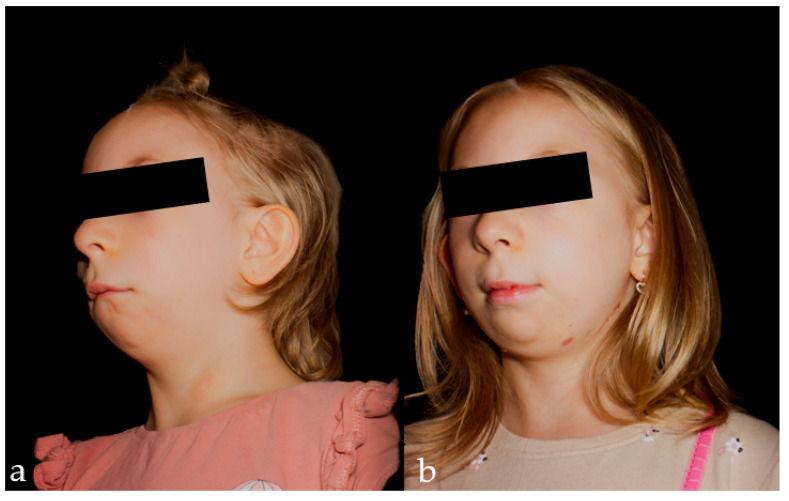
Extraoral photograph of the patient’s face, frontal view: (**a**) before surgery; (**b**) after removal of the distractors.

Mandibular DO is an effective technique for the correction of craniofacial deformities, particularly in pediatric patients with syndromic mandibular hypoplasia and associated airway obstruction. In this context, achieving precise osteotomy placement, a well-controlled distraction vector, and timely assessment of callus maturation are critical for optimizing functional and aesthetic outcomes. This case highlights the successful integration of 3D virtual planning and USG in a pediatric patient with first and second branchial arch syndrome, providing a model for personalized and radiation-conscious management of complex mandibular anomalies (Figure 9).

Preoperative planning using patient-specific 3D-printed models has gained increasing traction in maxillofacial surgery. Derived from CBCT scans, these models facilitate accurate visualization of the mandibular anatomy, allowing for tailored osteotomy design and vector orientation, particularly in cases involving anatomical asymmetry, unerupted teeth, or airway compromise. In our case, the ability to visualize and simulate osteotomies on a tangible 3D model proved essential in safely avoiding dental injury. Previous studies have confirmed that preoperative simulations using 3D-printed models can significantly reduce operative time, improve surgical accuracy, and enhance intraoperative decision-making, particularly in pediatric patients who are more vulnerable to prolonged anesthesia exposure [10,11,12].

Distraction vector orientation is one of the most important determinants of the success of DO, as it directly influences the morphology of the regenerate, the stability of occlusion, and airway dimensions [13]. Improper vector planning can result in inadequate airway improvement or relapse. In syndromic micrognathia, such as in Pierre Robin sequence or hemifacial microsomia, horizontal vector advancement is typically preferred to maximize airway patency while maintaining condylar position [14,15]. In our case, the preoperative model enabled the planning of a bilateral horizontal vector to symmetrically expand the mandible anteriorly, which contributed to improved breathing outcomes and occlusal alignment.

Intraoperative application of the planned vector was facilitated by piezoelectric osteotomy, which offers enhanced precision and reduced risk of thermal or soft tissue damage compared to conventional burs or saws [16]. Studies have shown that piezoelectric devices improve bone healing and reduce postoperative complications due to their selective cutting and minimal trauma to adjacent neurovascular structures [16].

Postoperative monitoring of distraction and bone regeneration represents another critical phase in DO management. Traditionally, serial panoramic radiographs or CT scans have been employed to evaluate callus formation and device positioning. However, cumulative radiation exposure poses significant long-term risks in pediatric populations, particularly when multiple follow-ups are required over extended periods [6,17]. In this context, B-mode ultrasonography has emerged as a valuable, radiation-free alternative for assessing distraction gaps, callus formation, and vascularity. Our findings confirm previous studies demonstrating that USG enables early detection of mineralization, cortical bridging, and callus maturation [7,17]. Doppler imaging can additionally confirm intraregional vascularity, serving as a proxy for bone viability and regenerative potential. In our case, USG clearly identified the osteotomy gap, monitored callus progression, and allowed confident decision-making for the timing of distractor removal.

### 4.1. Limitations

Limitations of this case report include the lack of a comparator or blinded assessment, limiting generalizability and inviting observer bias. Follow-up was short, and standardized pre- and post-treatment functional outcomes (e.g., repeat polysomnography, quantitative airway measurements, and validated patient- or parent-reported sleep and feeding questionnaires) were not collected, limiting our ability to formally quantify symptomatic improvement. Ultrasonography itself also has its limitations. It is operator-dependent, and image interpretation requires familiarity with bone healing patterns and device artifacts [6]. USG measurements are vulnerable to probe obliquity, soft-tissue pressure, and distractor-related artifacts. Our scanning protocol (probe, landmarks, timing, thresholds) reflects local practice and may not translate without validation. Therefore, the standardization of scanning protocols, including probe orientation and frequency selection, is essential to ensure consistency and reproducibility. Moving forward, training for maxillofacial surgeons or collaboration with radiologists experienced in musculoskeletal ultrasound may enhance diagnostic accuracy. Future prospective series should predefine USG landmarks and decision thresholds, report intra-/inter-rater reliability, pair imaging with objective clinical outcomes, and extend the follow-up period.

Despite these limitations, this case underscores the feasibility of integrating 3D technology and USG into a unified workflow for mandibular DO. By combining patient-specific anatomical modeling with non-invasive follow-up imaging, clinicians can reduce intraoperative uncertainty, limit radiation burden, and support more informed, timely postoperative decisions. Such approaches align well with precision medicine principles and are particularly suited to pediatric craniofacial surgery.

### 4.2. Future Prospects

Looking ahead, the integration of artificial intelligence (AI) into craniofacial surgery holds significant promise. AI-based tools are increasingly being developed to automate anatomical landmark detection and segmentation and to assist in virtual surgical planning. In the context of DO, AI has the potential to support decision-making regarding distraction vector planning, growth forecasting in pediatric patients, and follow-up monitoring. While these technologies are still in early stages of clinical translation, their integration with 3D imaging and ultrasonography could further personalize treatment, reduce variability, and enhance surgical precision in complex craniofacial reconstructions.

## 5. Conclusions

In conclusion, this case illustrates a radiation-conscious workflow in which patient-specific 3D modeling is combined with structured ultrasonographic monitoring to guide mandibular DO in a pediatric patient with first and second branchial arch syndrome and airway obstruction. Ultrasonography, as a non-invasive, radiation-free tool, enabled real-time assessment of callus maturation, cortical bridging, and vascularity and, together with panoramic radiography, informed the timing of distraction termination and distractor removal at 16 weeks. Although the observations derive from a single case, they suggest that integrating 3D virtual planning and serial US examinations may enhance the accuracy, safety, and individualization of DO protocols in children with complex mandibular deformities.

## Figures and Tables

**Table 1:** Summary of clinical and imaging findings during mandibular distraction osteogenesis.

Time Postoperatively	Osteotomy Gap Measurement	Ultrasonographic Findings	Radiographic Findings	Clinical Findings
Day 1	N/A	Correct positioning of distractors	Correct positioning of distractors	Patient monitored in ICU, no complications
Day 4—first day of distraction	N/A	N/A	N/A	Patient clinically stable
Day 6	2.5 mm	Early callus formation with good vascularization	None	No signs of infection
Day 21	15 mm	Progression of callus without complications	Initial calcification observed	Patient clinically stable
Day 42	13 mm	Progression of callus and mineralization	Continued calcification and cortical continuity observed	Patient clinically stable

N/A: not available; ICU: intensive care unit. Representative panoramic radiographs and ultrasonographic images for days 6, 21, 42 (week 6), and week 16 post-distraction are shown in Figure 5, Figure 7 and Figure 8.

## Data Availability

No new data were created.
